# Inhibition of NF-κB improves the stress resistance and myogenic differentiation of MDSPCs isolated from naturally aged mice

**DOI:** 10.1371/journal.pone.0179270

**Published:** 2017-06-22

**Authors:** Jonathan D. Proto, Aiping Lu, Akaitz Dorronsoro, Alex Scibetta, Paul D. Robbins, Laura J. Niedernhofer, Johnny Huard

**Affiliations:** 1 Department of Medicine, Division of Molecular Medicine, Columbia University, New York, NY, United States of America; 2 Department of Orthopaedic Surgery, Institute of Molecular Medicine, University of Texas Health Science Center at Houston, McGovern Medical School, Houston, TX, United States of America; 3 Center for Regenerative Sports Medicine, Steadman Philippon Research Institute, Vail, CO, United States of America; 4 Department of Metabolism and Aging, The Scripps Research Institute, Jupiter, FL, United States of America; University of Minnesota Medical Center, UNITED STATES

## Abstract

A decline in the regenerative capacity of adult stem cells with aging is well documented. As a result of this decline, the efficacy of autologous stem cell therapies is likely to decline with increasing donor age. In these cases, strategies to restore the function of aged stem cells would have clinical utility. Globally, the transcription factor NF-κB is up-regulated in aged tissues. Given the negative role that NF-κB plays in myogenesis, we investigated whether the age-related decline in the function of muscle-derived stem/progenitor cells (MDSPCs) could be improved by inhibition of NF-κB. Herein, we demonstrate that pharmacologic or genetic inhibition of NF-κB activation increases myogenic differentiation and improves resistance to oxidative stress. Our results suggest that MDSPC “aging” may be reversible, and that pharmacologic targeting of pathways such as NF-κB may enhance the efficacy of cell-based therapies.

## Introduction

Regenerative medicine and tissue engineering have traditionally focused on stem cell based approaches for the treatment of disease and injury. At present, clinical trials have taught us that many obstacles remain to be overcome before cell therapies can become practical therapeutic options. One problem is the impact of donor age on stem cell function. There is substantial evidence that stem cell function declines with age and this contributes to the aging process [1–5]. Thus, transplantation of older, functionally impaired cell populations results in a reduced therapeutic efficacy compared to the transplantation of younger cell populations [6–8]. This is an issue that needs to be addressed given that diseases for which stem cell-based treatments seem the most promising, such as cardiovascular and neurodegenerative diseases, are age-related [2, 9]. Considering that the risks associated with immunosuppression may outweigh the benefits of allogeneic cell transplants, new strategies aimed at improving aged stem cell function must be identified.

Molecules that target specific age-related pathways may improve adult stem cell regenerative capacity. Of the many pathways that have been implicated in aging, increased NF-κB activity has been identified as a major regulator of gene expression programs associated with aging across diverse tissues [10–12]. For example, increased amounts of NF-κB subunits have been found in nuclear extracts of aged mouse and rat skin, liver, kidneys, and brain [13]. Genetic inhibition of NF-κB in aged murine skin leads to tissue rejuvenation and changes in gene expression resembling younger skin [12]. Moreover, systemic inhibition of NF-κB, either by deletion of one allele of the NF-κB subunit p65 or by chronic administration of an inhibitor of the kinase upstream of NF-kB, delays aging in *Ercc1*^*-/Δ*^ mice, a model of XFE progeroid syndrome [14]. NF-κB activation is also implicated as a barrier to induced pluripotency in fibroblasts from aged patients, where p65 induces the transcription of *Dot1l*, a histone H3 methyltransferase that represses pluripotency-related genes [15].

This led us to hypothesize that NF-κB may represent a molecular target for enhancing the regenerative potential of muscle-derived stem/progenitor cells (MDSPCs). Previous studies demonstrated that MDSPC-based therapies promote tissue repair following muscle, bone, cartilage, and cardiac injury [16–19]. However, MDSPC function is impaired with age [4, 20]. For this investigation, we tested whether MDSPCs, isolated from 24 month-old mice, can be “rejuvenated” by pharmacologic means. We found that treatment with a small molecule inhibitor of NF-κB restored the myogenic capacity of aged MDSPCs to the same level as young MDSPCs, isolated from 14 day-old mice. Similar treatment of senescent bone marrow-derived mesenchymal stem cells reversed senescence. Furthermore, pretreatment of aged MDSPCs with an IKKβ/NF-κB inhibitor increased cell survival under oxidative stress. Similarly, we found that aged *p65*^+/-^ MDSPCs retained myogenic potential *in vitro* and had a higher resistance to oxidative stress-induced cell death than aged wild-type (WT) cells. Although NF-κB inhibition enhanced aged MDSPC differentiation and stress resistance, it did not improve cell proliferation. Instead, we were able to increase proliferation using three other compounds previously reported to extend murine lifespan [21, 22]. These results demonstrate that *ex vivo* rejuvenation of aged MDSPC function is feasible. Pharmacologic treatment may represent one strategy for enhancing the efficacy of autologous cell therapies in aging patients.

## Materials and methods

### Reagents

IKK-2 Inhibitor IV and VII was obtained from EMD Millipore (MA, USA). Aspirin, nordihydroguaiaretic acid, and rapamycin were obtained from Sigma-Aldrich (PA, USA).

### Cell isolation

Populations of muscle-derived cells were isolated from the leg muscles of 24 month-old (aged) and 2 week-old (young) WT mice, and 30 month-old p65 haploinsufficient (aged *p65*^+/-^) mice (all F1 C57BL/6:FVB) using the previously described modified pre-plate technique [23]. MDSPCs were collected and expanded in growth medium consisting of 10% FBS, 10% horse serum, 1% Penn-strep, and 0.5% chick embryo extract (Accurate Chemical Co., New York, USA) in DMEM. Mesenchymal stem cells (MSCs) were obtained from the bone marrow (BM) of 23 week-old mice (mature adult) and cultured in high glucose DMEM supplemented with 15% FBS, 2mM glutamine, 100 U/ml penicillin and 0.1 mg/ml streptomycin (all from Sigma-Aldrich, St Louis, MO, USA). The MSCs were cultured in low oxygen conditions (3% O_2_) to avoid oxidative damage. In brief, the BM cells were flushed out from the long bones using a syringe following euthanization of the mice. The extracted BM cells were washed by centrifugation and seeded to a concentration of 2.5x10^5^ cells/cm^2^, while considering this passage 0. Non-adherent cells were discarded by changing the media 16h later and the culture was further enriched for MSCs by culturing the BM cells for 3 passages. The generated MSCs displayed a CD105+, CD106+, CD73+, Sca-1+, CD34-, CD45- and CD31- phenotype, fibroblast-like morphology and multilineage differentiation capacity. All experiments were approved by the University of Pittsburgh or The Scripps Research Institute Institutional Animal Care and Use Committees.

### Myogenic differentiation assay and fast myosin heavy chain (MyHC) staining

After 15 passages, MDSPCs were plated on 24 well plates (20,000 cells per well) in DMEM supplemented with 2% FBS to stimulate myotube formation. Aged murine MDSPCs were treated with IKK-2 Inhibitor IV (EMD Millipore) at varying doses during myogenic differentiation to inhibit NF-κB activation. At the indicated time points, cells were washed, fixed with ice cold 100% methanol, and stained for fast skeletal MyHC. Briefly, cells were blocked with 10% horse serum for 1 h and then incubated with a mouse anti-MyHC (1:250, Sigma-Aldrich) for 1 h at room temperature. Cell bound primary antibody was detected with a secondary anti-mouse antibody conjugated with AlexaFluor594 (1:500, Sigma-Aldrich) by incubation for 30 min. The nuclei were revealed by DAPI staining. The percentage of differentiated myotubes was quantified as the number of nuclei in MyHC positive myotubes relative to the total number of nuclei.

### *In vitro* measurement of cell survival under oxidative stress

MDSPCs were exposed to oxidative stress induced by treatment with 250 uM hydrogen peroxide. In order to visualize cell death, propidium iodide (PI), a DNA-binding dye, was added to culture medium according to the manufacturer’s protocols (BD Bioscience, CA, USA). Using a previously described live cell imaging system (LCI; Kairos Instruments LLC, Pittsburgh, PA) [24], 10x bright field and fluorescence images were taken in 10 minute intervals over 24 h [24]. Identifying the number of PI+ cells per field of investigation out of the total cell number determined the percentage of cell death over time.

### Measurement of stem cell senescence

MSC senescence was determined by measurement of senescence specific β-galactosidase activity (SA β-gal). Cells were seeded at 5,000 cell/cm^2^ and treated for 48 h with IKK-2 inhibitor VII (600 nM, EMD Millipore). Then cells were fixed with 2% PFA for 5 min and stained overnight with X-gal staining buffer, pH 6, containing 2 mg/ml of X-gal (Teknova, Hollister, CA, USA) at 37°C. The nuclei were labeled with DAPI (Life technologies, Carlsbad, CA, USA) and the SA β-gal+ cells were counted manually.

### Cell proliferation

Using the LCI system described above, bright field images (10x) were taken at 10 minute intervals over a 72-hour period in three fields of view per well, with three wells per population. Using ImageJ software (NIH), proliferation was assessed by counting the number of cells per field of view over 12 hour intervals.

### Statistical analysis

All results are given as the mean ± standard error of the mean. Means were compared using the student’s T-test or ANOVA with Tukey’s post hoc analysis, as appropriate. Differences were considered statistically significant when the p-value was <0.05.

## Results

### Inhibition of NF-κB/IKKβ restores the myogenic potential of aged murine MDSPCs

**Fig 1:**
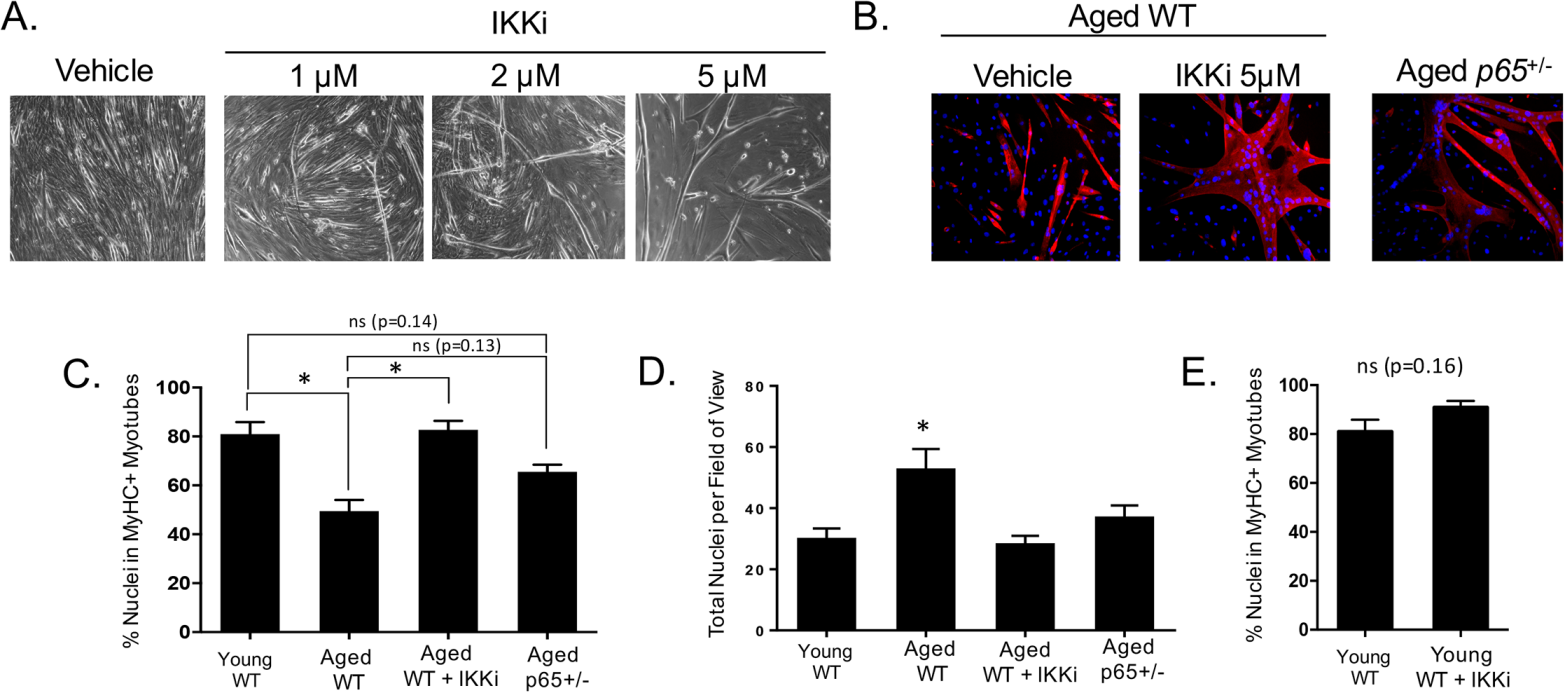
NF-κB inhibition improves the differentiation of MDSPCs isolated from old mice. (A) Brightfield images demonstrate a dose dependent increase in myotube formation following IKKi administration (10x images). (B) Immunofluorescent staining for MyHC demonstrated significantly improved differentiation following treatment at the 5μM dose (20x magnification), quantified in (C). (D) The total number of nuclei per field of view for experiment in B, C (*p≤0.05; ANOVA followed by Tukey post-hoc analysis). (E) IKKi treatment of young WT MDSPCs did not significantly improve differentiation (*p≤0.05, T-test).

We investigated whether NF-κB inhibition would be sufficient to restore the myogenic potential of aged MDSPCs. Cells were isolated from young (14 day-old) and aged (24 month-old) mice via the modified preplate technique [23]. Aged and young MDSPCs were cultured under myogenic conditions with or without the presence of IKK-2 inhibitor IV (IKKi), an inhibitor of the NF-κB activating kinase, IKKβ, at varying doses. Myogenic differentiation was monitored by bright field microscopy, and after 5 days, treated cells demonstrated an increase in myotube formation (Fig 1A). We quantified differentiation of myocytes at 5 μM IKKi (the most effective dose) by immunofluorescent staining for fast skeletal MyHC, a terminal marker of skeletal muscle differentiation (Fig 1B and 1C). Strikingly, IKKi treatment of aged MDSPCs increased myogenic differentiation to levels similar to those of young cells. Although all groups were plated at the same density, after 5 days of culture under myogenic conditions, the total number of nuclei per field of view was increased in the untreated (vehicle control) aged MDSPC group (Fig 1D). Interestingly, this was normalized by IKKi treatment. This is in agreement with a previous report that NF-κB activity can inhibit differentiation by delaying exit from the cell cycle [25]. To check for off-target effects of IKKi, we also assessed the myogenic capacity of MDSPCs haploinsufficient for the NF-kB subunit p65 (*p65*^+/-^) isolated from 30 month-old mice. Unlike aged WT cells, *p65*^+/-^ MDSPCs from aged mice did not demonstrate a significant decline in differentiation compared to young WT cells (p = 0.14, Fig 1C). IKKi treatment did not enhance differentiation of young WT MDSPCs, indicating this effect was specific to aged cells (Fig 1E). Taken together, these results demonstrate that IKK/NF-κB inhibition restores the myogenic differentiation potential of aged MDSPCs.

### NF-κB inhibition increases MDSPC resistance to hydrogen peroxide induced oxidative stress

**Fig 2:**
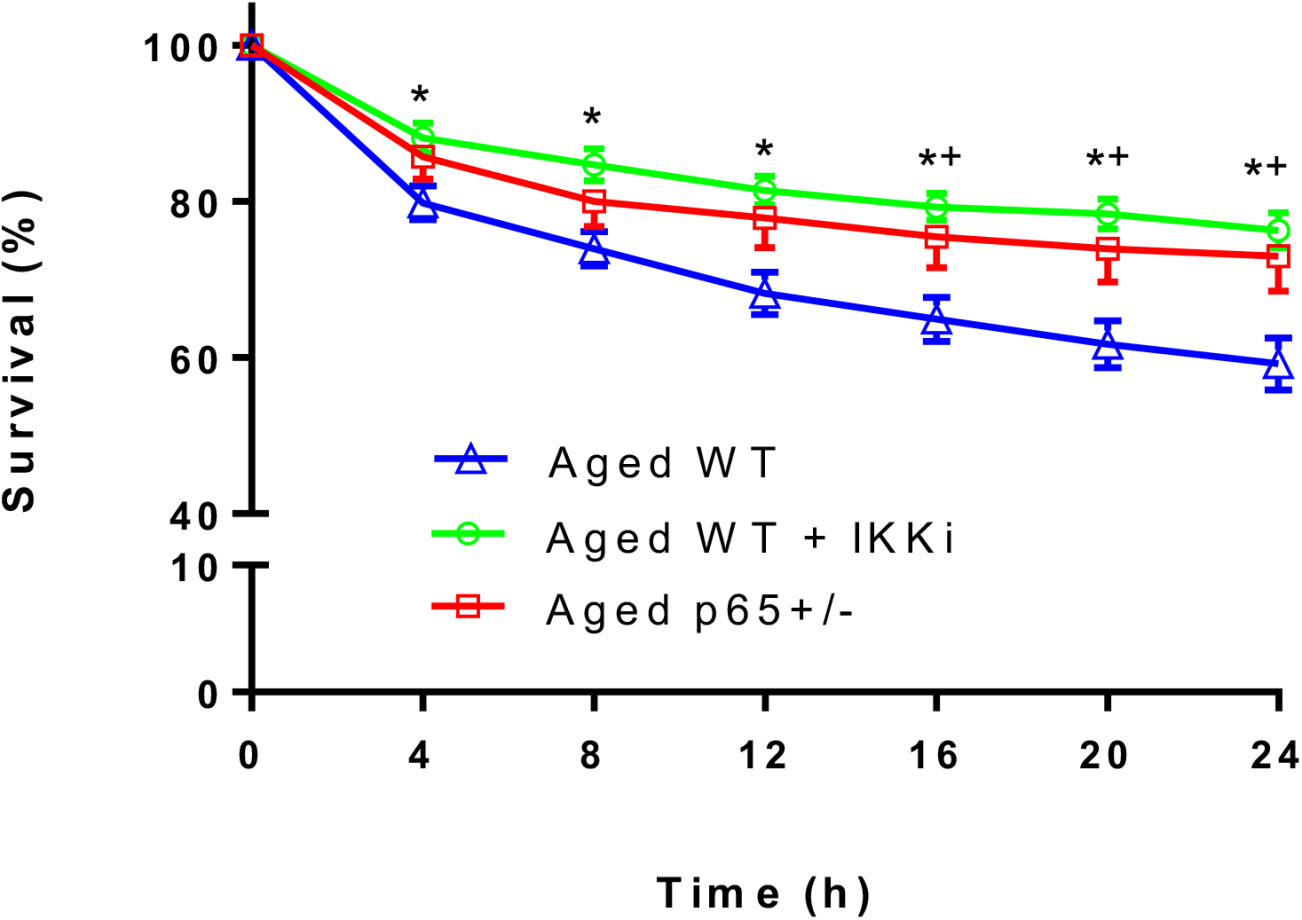
NF-κB inhibition improves the oxidative stress resistance of MDSPCs isolated from old mice. MDSPCs were exposed to 250 μM H_2_O_2_ and monitored by live cell imaging for up to 24 hours. IKKi treated aged WT MDSPCs and aged *p65*^+/-^ MDSPCs demonstrated improved survival compared to vehicle treated aged WT MDSPCs (*p≤0.05 Aged WT vs +IKKi; +p≤0.05 Aged WT vs Aged *p65*^+/-^; ANOVA followed by Tukey post-hoc analysis).

Previous studies from our group demonstrated that MDSPC resistance to oxidative stress, which declines with donor age, is an important characteristic that distinguishes them from committed myoblasts and correlates with high regenerative capacity *in vivo* [4, 26]. As NF-κB is known to be a stress response pathway [27, 28], we examined the effect of IKK/NF-κB inhibition on cell survival in resppnse to hydrogen-peroxide (H_2_O_2_) induced oxidative stress. Briefly, cells were exposed to H_2_O_2_ in proliferation medium containing PI, a fluorescent DNA stain. Cell survival was measured by using live cell imaging (LCI) for up to 24 h. Pretreatment of the cells with IKKi significantly increased survival of MDSPCs isolated from aged mice. Likewise, genetic depletion of p65 improved MDSPC oxidative stress resistance (Fig 2).

### Drugs that extend the lifespan of mice promote proliferation of MDSPCs

**Fig 3:**
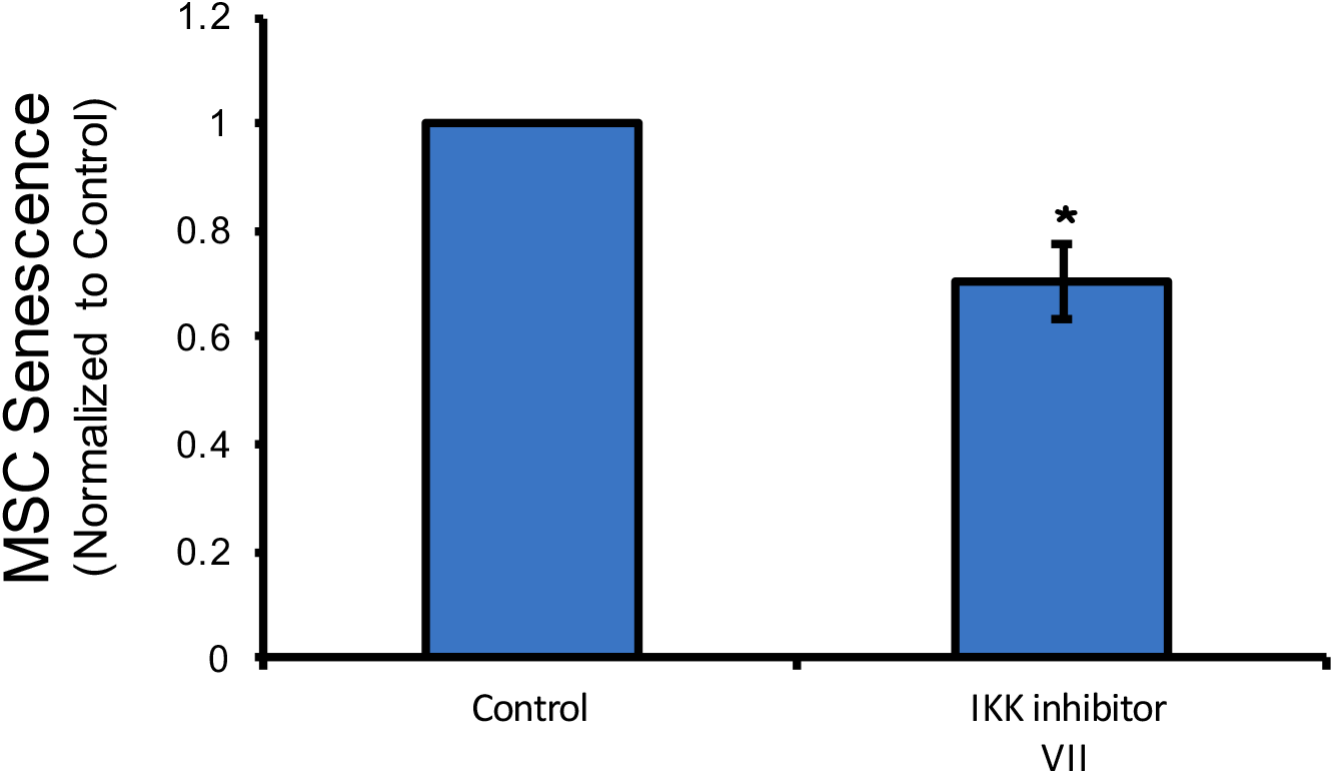
NF-κB inhibition decreases senescence in BM-MSCs. BM-MSCs were treated for 48 hours with IKK inhibitor VII and then stained for senescence-associated beta-galactosidase (SA β-gal) expression, which demonstrated a decrease in cell senescence (*p≤0.05, T-test).

**Fig 4:**
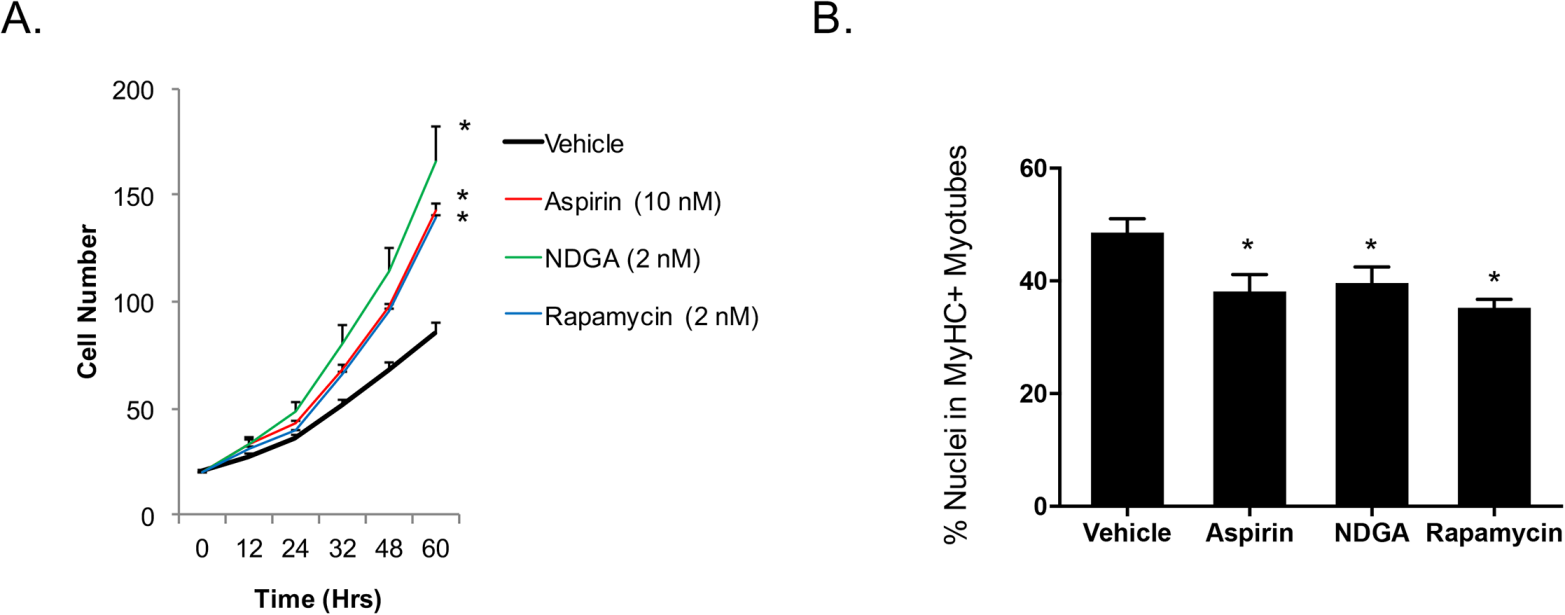
Multiple compounds improve aged MDSPC proliferation (self-renewal). (A) Aged MDSPCs were cultured in medium containing aspirin, nordihydroguaiaretic acid (NDGA), or rapamycin, and proliferation was monitored by live cell imaging for up to 60 hours, or (B) cells were treated in fusion medium and assessed for myogenic differentiation after 5 days (*p≤0.05 Aged WT vs Treated; ANOVA followed by Tukey post-hoc analysis).

Although IKK/NF-κB inhibition improved myogenic differentiation and stress resistance, it had the opposite effect on cell proliferation. However, we cannot exclude the possibility that the enhanced myogenic differentiation did not indirectly contribute to the effect of IKK/NF-κB inhibition on cell proliferation, as cell cycle withdrawl preceeds terminal differentiation[29]. Interestingly, we found that after 48 h of IKK/NF-κB inhibition, senescence of bone marrow-derived mesenchymal stem cells (BM-MSCs) was reduced, as assessed by beta-galactosidase positivity, a commonly used marker of senesence [30, 31] (Fig 3). This suggests that NF-κB may play a role in stem cell self-renewal and senescence in at least BM-MSCs. To determine if other lifespan extending interventions, in addition to inhibiting NF-κB activation [14], improve adult stem cell function, we tested the effect of several hits from the National Institute on Aging (NIA) Intervention Testing Program (ITP) [32] for their ability to increase MDSPC self-renewal. We tested a number of compounds reported to extend lifespan in mice, including aspirin, the antioxidant nordihydroguaiaretic acid (NDGA) [21], and the mTOR inhibitor rapamycin [22]. All three significantly increased proliferation (self-renewal) of MDSPCs isolated from aged mice (Fig 4A). We also measured the effect of these compounds on aged MDSPC myogenic differentiation. In contrast to NF-kB inhibition, which did not promote proliferation but did increase differentiation, aspirin, NDGA, and rapamycin all decreased differentiation (Fig 4B). Considering that proliferation and differentiation are mutually exclusive, this result is not surprising. Based on these data, different pharmacologic agents likely need to be used to first expand and then differentiate stem cells isolated from aged individuals.

## Discussion

A decline in the function of tissue-resident stem cells is likely to contribute to the decreased wound healing and tissue regeneration that comes with age [1]. For example, skeletal muscle injury that would lead to the regeneration of functional tissue in children, instead leads to prolonged inflammation and fibrosis in older adults [33]. In skeletal muscle, this phenomenon is partially related to the functional decline of satellite cells, the skeletal muscle stem cell. In landmark experiments, the dysfunction of murine satellite cells isolated from aged mice was rescued by exposure to young mouse serum [34, 35]. This suggests that age-associated stem cell decline is reversible and may be due to signals from the aged tissue environment (niche). Thus, *ex vivo* priming to “rejuvenate” cells may be a viable approach for enhancing the function of cell-based therapies in the elderly [1].

Here we tested whether the phenotype of murine MDSPCs isolated from aged organisms could be improved by pharmacologic means. We previously described the anti-myogenic role of NF-κB in MDSPC differentiation [36], and the mechanisms through which NF-κB blocks myoblast differentiation have been thoroughly investigated [25, 37]. Furthermore, a recent report indicates that NF-κB activation in aged skeletal muscle fibers reduces satellite cell myogenic differentiation potential [38]. Indeed, we identified the NF-κB pathway as a potent target to restore the myogenic differentiation potential of aged MDSPCs. We also found that resistance to oxidative stress, an important stem cell characteristic [39], was also elevated in both *p65* deficient and IKKi-treated aged cells. This is in line with a previous report in which we found that donor *p65*^*+/-*^ MDSPCs have enhanced survival *in vivo* following transplantation to injured host skeletal muscle [40]. Furthermore, in a mouse model of accelerated aging, blockade of NF-κB reduced oxidative stress-induced DNA damage [14]. Based on our data, NF-κB activation in aged cells in response to oxidative stress might predominantly act in a pro-death or pro-cell senescence manner. Although NF-κB is traditionally considered a survival factor, under some circumstances, such as DNA damage-triggered apoptosis, a pro-apoptotic role for NF-κB has been well documented [41–43]. Further investigation will be necessary to determine if this is the case during oxidative stress-induced MDSPC death.

Although targeting NF-κB did not improve MDSPC proliferation, we identified three other compounds, NDGA, rapamycin, and aspirin that promote MDSPC self-renewal *ex vivo*. In large, multicenter studies, all three of these compounds extend the lifespan of male mice [21, 22]. In support of our findings, rapamycin prevents senescence of aged hematopoietic stem cells during *ex vivo* expansion [44], reduces MDSPC senescence, and improves myogenic differentiation of progeroid MDSPCs [45]. Interestingly, aspirin has been reported to promote endothelial progenitor cell migration and delay senescence, while having no effect on proliferation [46]. The effects of NDGA on stem cells are less understood. Future studies will be required to determine the exact mechanisms through which these compounds exert their effects on MDSPCs and whether treatment *ex vivo* will improve stem cell function *in vivo*. Nonetheless, our data indicate that pharmacologic strategies can improve stem cell function *in vitro*, and such an approach may prove useful to enhance cell therapies aimed at an aging patient population.
